# Optimizing strategies to identify high risk of developing type 2 diabetes

**DOI:** 10.3389/fendo.2023.1166147

**Published:** 2023-06-28

**Authors:** Paula Andreghetto Bracco, Maria Inês Schmidt, Alvaro Vigo, José Geraldo Mill, Pedro Guatimosim Vidigal, Sandhi Maria Barreto, Mária de Fátima Sander, Maria de Jesus Mendes da Fonseca, Bruce Bartholow Duncan

**Affiliations:** ^1^ Postgraduate Program in Epidemiology, School of Medicine, Universidade Federal do Rio Grande do Sul, Porto Alegre, Brazil; ^2^ Institution of Mathematics and Statistics, Universidade Federal do Rio Grande do Sul, Porto Alegre, Brazil; ^3^ Hospital de Clínicas de Porto Alegre, Porto Alegre, Brazil; ^4^ Health Science Center, Universidade Federal do Espírito Santo, Vitória, Brazil; ^5^ School of Medicine, Universidade Federal de Minas Gerais, Belo Horizonte, Brazil; ^6^ Clinical Hospital/EBSERH, Universidade Federal de Minas Gerais, Belo Horizonte, Brazil; ^7^ National School of Public Health, Oswaldo Cruz Foundation, Rio de Janeiro, Brazil

**Keywords:** type 2 diabetes, screening strategies, screening tool, mass screening, prediction score, sensitivity, positive predictive value

## Abstract

**Introduction:**

The success of diabetes prevention based on early treatment depends on high-quality screening. This study compared the diagnostic properties of currently recommended screening strategies against alternative score-based rules to identify those at high risk of developing diabetes.

**Methods:**

The study used data from ELSA-Brasil, a contemporary cohort followed up for a mean (standard deviation) of 7.4 (0.54) years, to develop risk functions with logistic regression to predict incident diabetes based on socioeconomic, lifestyle, clinical, and laboratory variables. We compared the predictive capacity of these functions against traditional pre-diabetes cutoffs of fasting plasma glucose (FPG), 2-h plasma glucose (2hPG), and glycated hemoglobin (HbA1c) alone or combined with recommended screening questionnaires.

**Results:**

Presenting FPG > 100 mg/dl predicted 76.6% of future cases of diabetes in the cohort at the cost of labeling 40.6% of the sample as high risk. If FPG testing was performed only in those with a positive American Diabetes Association (ADA) questionnaire, labeling was reduced to 12.2%, but only 33% of future cases were identified. Scores using continuously expressed clinical and laboratory variables produced a better balance between detecting more cases and labeling fewer false positives. They consistently outperformed strategies based on categorical cutoffs. For example, a score composed of both clinical and laboratory data, calibrated to detect a risk of future diabetes ≥20%, predicted 54% of future diabetes cases, labeled only 15.3% as high risk, and, compared to the FPG ≥ 100 mg/dl strategy, nearly doubled the probability of future diabetes among screen positives.

**Discussion:**

Currently recommended screening strategies are inferior to alternatives based on continuous clinical and laboratory variables.

## 1 Introduction

The effectiveness of early treatment to prevent diabetes (1, 2) has led medical associations and expert groups to recommend screening (3, 4) and countries to initiate national diabetes prevention programs (5–7). However, defining high risk is challenging. Recommended definitions are traditionally based on one or more established cutoffs of glycemic tests (3, 8, 9). Screening questionnaires have also been applied alone or together with laboratory results (5, 10–12). Clinical prediction scores have also been developed (13–16). However, direct, head-to-head comparisons of the diagnostic metrics of more sophisticated prediction score-based approaches with those of nationally recommended screening strategies are absent.

Our objective was to validate and compare diagnostic metrics of several screening strategies—those currently recommended in three countries with national screening programs (the United States, the United Kingdom, and Finland) and score-based strategies to detect high-risk individuals for primary diabetes prevention.

## 2 Methods

### 2.1 Study design, study population, and ethics approval

The ELSA-Brasil cohort study enrolled 15,105 public servants aged 35–74 between 2008 and 2010 (17) and conducted two return evaluations in 2012–2014 and 2016–2018 (18). The Research Ethics Committees approved the study protocol at each investigation site, and all participants gave written consent. Using standardized questionnaires and protocols, we obtained sociodemographic and clinical data (hereafter denominated “clinical variables”) (19, 20).

We ascertained diabetes at baseline and follow-up visits by self-report, antidiabetic medication use, and three laboratory measures—fasting plasma glucose (FPG) ≥ 126 mg/dl (7 mmol/L), 2-h plasma glucose (2hPG) ≥ 200 mg/dl (11.1 mmol/L) in a standard oral glucose tolerance test (OGTT), and glycated hemoglobin (HbA1c) ≥6.5% (48 mmol/mol). We considered a prevalent case at baseline when any of these criteria were present. To be consistent with clinical recommendations, we required confirmation of our incident diabetes cases. We thus ascertained incident diabetes only if at least one of these five criteria were present at both follow-up visits or at least two were found at a single visit. Research staff ascertaining diabetes at follow-up were unaware of baseline laboratory values. We considered those who met two criteria at the first follow-up but none at the second follow-up as not having developed diabetes. We excluded those without data for incident diabetes due to death, who lack follow-up, or with incomplete data from analyses.

### 2.2 Recommended screening strategies evaluated

We first assessed screening strategies based on traditional laboratory cutoffs for pre-diabetes (intermediate hyperglycemia) (3, 8, 21). Next, we evaluated additional screening recommendations used in national diabetes prevention programs. In addition to the United States, the United Kingdom and Finland have ongoing national programs (5, 12, 22). The American Diabetes Association (ADA) recommends two screening options (3). Considering that our sample begins at age 35, the first is a one-step (test all) approach directly measuring glycemia or HbA1c. The second applies a two-step approach in which those positive on a questionnaire are considered at high risk if they present FPG ≥ 100 mg/dl or HbA1c ≥ 5.7% (39 mmol/mol) in subsequent testing (3). The Centers for Disease Control and Prevention (CDC) recommends screening with either the ADA approach or just the ADA questionnaire (22). For the United Kingdom, the National Institute for Health and Care Excellence (NICE) recommends a two-step strategy—a clinical score followed by lab testing for those above its cutoff (FPG ≥ 99 mg/dl or an HbA1c ≥ 6.0% [42 mmol/mol]) (12). The Finnish strategy considers all those with a FINDRISC questionnaire score ≥15 as high risk (5, 23). Applying ELSA cohort follow-up data, we compared the diagnostic properties of these strategies with those of the scored-based screening strategies that we developed.

### 2.3 Statistical analysis

We randomly divided our sample equally into training and validation datasets. We described our sample characteristics calculating either the mean (standard deviation) or mean (95% confidence interval) for continuous variables and the absolute frequency (percentage; 95% confidence interval) for discrete variables. We used logistic regression on the training dataset to develop risk functions to predict incident diabetes. We initially produced models only with clinical variables, keeping those that significantly improved the area under the receiver operating characteristic (AUC) curve. We considered age, sex, self-reported ethnicity, educational attainment, parental history of diabetes, daily consumption of fruits or vegetables, leisure-time physical activity (at least 150 min per week of moderate- or vigorous-intensity physical activity), smoking, hypertension or self-reported use of hypertension medication, body mass index (BMI), and waist circumference. We then selected the best of these derived risk functions to calculate the probability of developing diabetes for each participant in our validation dataset. Using this same approach, we next evaluated risk scores composed of laboratory results and their combination with the clinical data. We provide risk function formulas in the Supplementary Material. Additionally, we built an online tool using R Shiny (http://elsabrasil.org/funcoes-de-risco/risco-diabetes-10-anos/) for risk calculation based on scores composed of different combinations of variables to predict the 10-year risk of developing diabetes to be made available to the public.

These risk scores, different from categorical rule approaches, for which cutoff points have already been defined (e.g., the ADA questionnaire ≥5; FPG ≥ 100 mg/dl) (3, 12, 22), have no *a priori* cutoff. To evaluate their properties, we thus defined potential cutoffs based on their positive predictive value (PPV), selecting ones where being positive reflected 20% and alternatively 15% probabilities of developing diabetes over our sample’s follow-up. These were close to the probabilities of developing the disease among those labeled by current ADA laboratory testing strategies.

Using the same ELSA database, we also constructed the clinical scores recommended by national strategies. As we lacked information for one of the FINDRISC questions—a previous finding of pre-diabetes—we randomly attributed the presence of prior pre-diabetes to 90% of those with baseline hyperglycemia by WHO criteria.

Finally, in our validation sample, we calculated diagnostic properties for the rules assessed: the percentage deemed at high risk, sensitivity, specificity, and positive and negative (1 − PPV) predictive values. We also report the AUC and the net reclassification index (16). We estimated 95% confidence intervals through normal approximation methods.

## 3 Results

**Figure 1:**
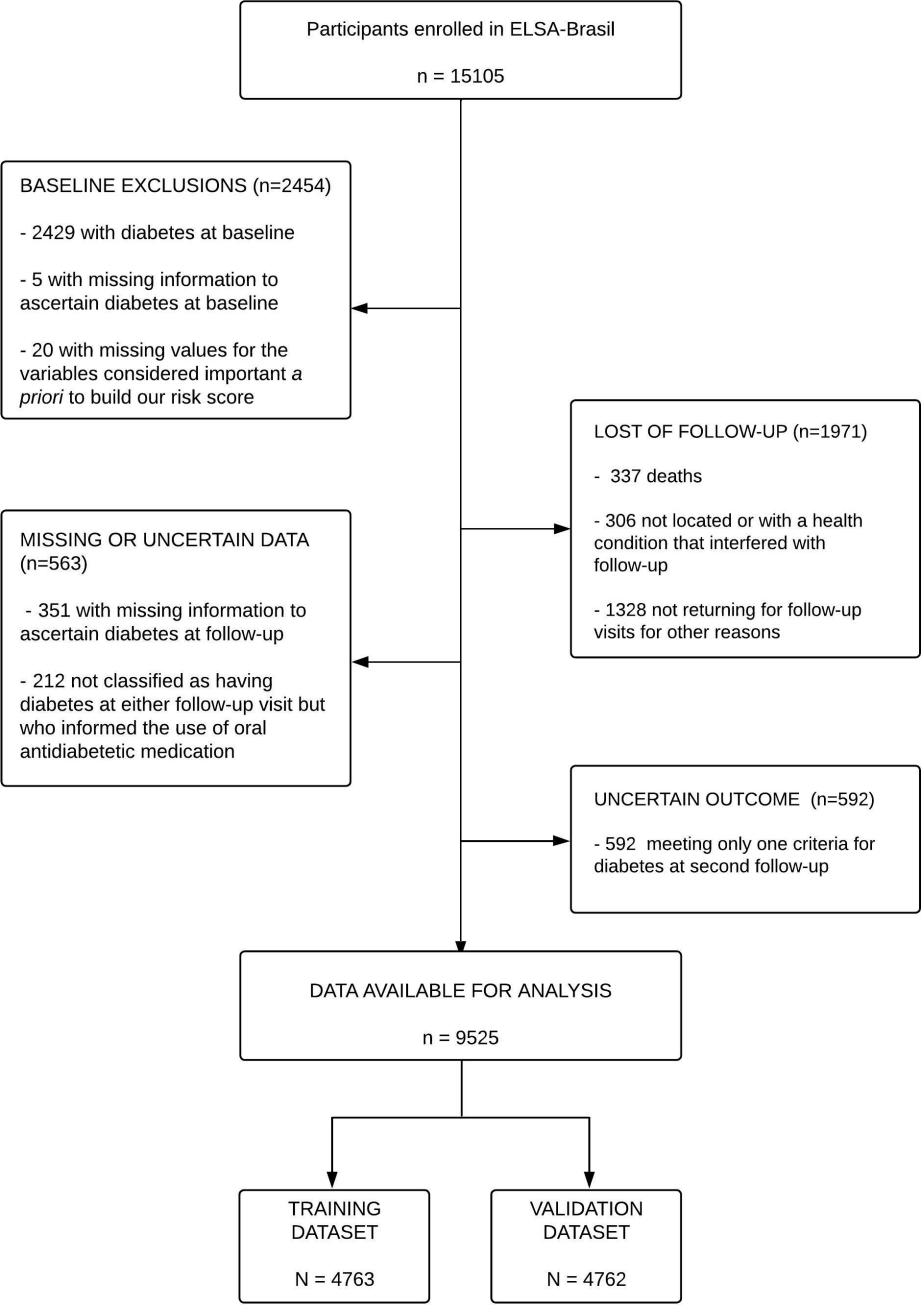
Flow diagram for the selection of the analytic sample.

Our study used data from the 15,105 participants of the ELSA-Brasil cohort. We excluded those with diabetes at baseline (n = 2,429), missing information to ascertain diabetes (n = 5), or missing values for variables we considered in the construction of risk scores (n = 20). We additionally excluded those not returning to follow-up visits (n = 1,971), missing data to ascertain incident diabetes (n = 351), or using oral antidiabetic medication but not reporting to have diabetes (n = 212). Finally, due to our requirement of confirmation of incident cases, we excluded those with no finding of diabetes at the first follow-up and only one criterion present at the second follow-up visit (n = 592). Our final sample thus consisted of 9,525 participants (**Figure 1**), randomly divided into training and validation samples.

Over a mean (standard deviation) of 7.4 (0.54) years of follow-up, 864 participants developed diabetes. We considered 24 participants who met two criteria for diabetes at the first follow-up but none at the second follow-up as not having developed diabetes. **Supplementary Table 1** shows that training and validation datasets had similar distributions of variables considered in building risk scores and a similar (9.1%) incidence of diabetes.

**Table 1** compares three cardinal metrics for screening evaluation—the percentage of screen positives (those labeled as high risk), sensitivity (percentage of future incident cases among screen positives), and PPV (percentage of screen positives who developed future diabetes). The complement of PPV (1 − PPV) also evaluates the false-positive rate. The top part of the table shows results for laboratory-based approaches. Since ELSA-Brasil participants were at least age 35 at entry, the one-step ADA laboratory option was to test all and consider positive those above the established laboratory cutoffs for pre-diabetes. If only FPG is tested, though a large percentage (76.6%) of those who developed diabetes were detected, a high percentage (40.6%) were labeled as high risk, most of them (1 − PPV; 82.9%%) not developing diabetes during our follow-up. Similar strategies testing FPG plus HbA1c or 2hPG produced similar results. The previous 2021 ADA recommendation of testing all ≥45 years of age and those younger when presenting specific conditions (evaluated here with only FPG testing) also performed similarly. It identified slightly fewer future cases (76.1% *vs.* 76.6%) while labeling marginally fewer (39.8% *vs.* 40.6%) as high risk with a slightly lower false-positive rate (82.7% *vs.* 82.9%). Testing all with the WHO fasting glucose cutoff of ≥110 mg/dl labeled considerably fewer participants (11.0%) as high risk and produced fewer false positives (1 − PPF; 67.0%) but identified a lower percentage of future cases (40.1%).

**Table 1:** Diagnostic properties of currently recommended one- or two-step categorical screening strategies in the ELSA-Brasil validation sample (N = 4762).

	High risk	Sens	PPV
**Laboratory only (one-step)**	% (95%CI)	% (95%CI)	% (95%CI)
Current ADA: FPG ≥ 100 mg/dl	40.6 (39.2; 42.0)	76.6 (72.6; 80.6)	17.1 (15.4; 18.7)
Current ADA: FPG ≥ 100 mg/dl or 2hPG ≥ 140 mg/dl	47.5 (46.1; 49.0)	86.7 (83.5; 89.9)	16.5 (15.0; 18.1)
Current ADA: FPG ≥ 100 mg/dl or HbA1c ≥ 5.7%	47.2 (45.8; 48.6)	82.3 (78.7; 85.9)	15.7 (14.2; 17.2)
Previous ADA (2021, all aged ≥45)*: FPG ≥ 100 mg/dl	39.8 (38.4; 41.2)	76.1 (72.1; 80.1)	17.3 (15.6; 19.0)
Current WHO: only FPG ≥ 110 mg/dl	11.0 (10.1; 11.9)	40.1 (35.5; 44.8)	33.0 (29.0; 37.0)
Current WHO: FPG ≥ 110 mg/dl or 2hPG ≥ 140 mg/dl	24.9 (23.6; 26.1)	70.2 (65.8; 74.5)	25.6 (23.1; 28.1)
**National program strategies**			
ADA questionnaire	18.5 (17.3; 19.6)	36.4 (31.8; 40.9)	19.1 (16.4; 21.7)
ADA two-step strategy	13.1 (12.1; 14.1)	34.0 (29.5; 38.5)	24.9 (21.4; 28.4)
NICE two-step strategy	26.8 (25.5; 28.1)	60.4 (55.7; 65.0)	21.3 (19.0; 23.6)
FINDRISC questionnaire	17.8 (16.7; 18.9)	41.5 (36.8; 46.2)	21.2 (18.4; 24.0)

Sens, sensitivity; PPV, positive predictive value; FPG, fasting plasma glucose; ADA, American Diabetes Association; WHO, World Health Organization; HbA1c, glycated hemoglobin; 2hPG, 2-h plasma glucose.

Lipids = triglycerides and high-density lipoprotein cholesterol. ADA questionnaire: risk score ≥5; ADA two-step strategy: risk score ≥5, then FPG ≥ 100 mg/dl or HbA1c ≥ 5.7%; NICE two-step strategy: U.K. Diabetes Risk Score ≥14, then FPG ≥ 99 mg/dl or HbA1c ≥ 6%; FINDRISC questionnaire: risk score ≥15.

*Those under age 45 presenting known risk factors were also tested.

In the lower portion of **Table 1**, we present and contrast diagnostic properties of additional strategies recommended by national screening programs. The ADA questionnaire alone (AUC = 0.599; 95%CI 0.576–0.623), one of the CDC’s recommended screening tests, labeled 18.5% as high risk, but with 80.9% (1 − PPV) of these being false positives while detecting only 36.4% of future cases of diabetes. Assuming a second step testing both FPG and HbA1c, the ADA two-step strategy presented a low AUC (0.615; 95%CI 0.592–0.638), labeled 13.1% as high risk with 75.1% false positives while detecting only 34.0% of those who went on to develop diabetes. Similarly, values for the NICE two-step approach, when combined with the Diabetes U.K. risk questionnaire (AUC = 0.685; 95%CI 0.661–0.710), labeled 26.8%, with 78.7% false positives and 60.4% of future cases detected. The FINDRISC questionnaire (AUC = 0.630; 95%CI 0.606–0.655) labeled 17.8%, with 78.8% false positives and 41.5% of future cases being detected. **Supplementary Table 2** presents an expanded array of diagnostic properties for these and other one-step laboratory testing strategies.

Next, we evaluated the clinical scores that we had developed. The best score is based only on readily available clinical variables of modeled risk as a function of age, sex, self-declared ethnicity, BMI, waist circumference, hypertension, and parental history of diabetes (AUC = 0.754; 95%CI 0.732–0.777). Further adjustment with other diabetes risk factors did not improve this score.

**Figure 2:**
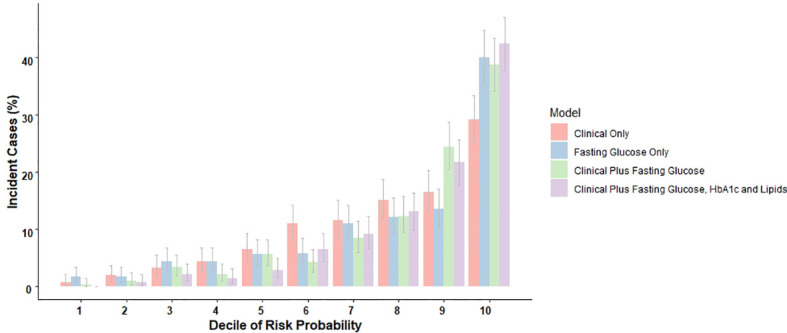
The distribution of incident cases during an average of 7.4 years of follow-up across deciles of risk, as predicted by rules with just clinical variables, just fasting glucose, and combinations of both with and without additional laboratory determinations. ELSA-Brasil validation sample, N = 4,762.

We additionally evaluated similarly derived prediction rules composed of laboratory tests, alone or combined with clinical variables, expressing these laboratory variables continuously rather than categorically based on recommended cutoffs. For these analyses, we initially defined high risk as a 20% probability of developing diabetes over our 7.4-year average follow-up. **Figure 2** illustrates the marked increase in the percentage of the ELSA-Brasil validation sample developing incident diabetes across risk deciles produced by four continuous variable strategies. Of note, all four prediction rules—clinical variables only, FPG only, clinical variables plus FPG, and clinical variables adding HbA1c and lipids along with FPG—distinguished those at minimal risk (risk close to 0%, first two deciles) from those at very high risk (35% to >40%, top decile). Scores combining clinical and laboratory tests placed more than 60% of those who developed diabetes in the top two deciles of estimated risk.

**Table 2** presents the same diagnostic properties above for several continuous variable rules, including those based on two-step approaches (clinical variable score first, then laboratory testing for those above an initial risk cutoff). With a score cutoff identifying a 20% probability of developing future diabetes, these rules labeled a considerably lower percentage of the sample—between 10.9% and 15.3%—as high risk than most nationally recommended strategies. That based only on clinical variables performed poorly, identifying only 31.6% of future cases. Adding FPG testing to clinical variables considerably improved future case detection to 49.7%, and further adding HbA1c, triglycerides, and HDL-c tests raised it to 54.5%. The two-step approach—laboratory testing only in those at highest risk based on clinical variables—reduced laboratory testing but with some loss in future case detection. For example, testing with FPG only the 50% or 67% at highest clinical risk (*vs.* testing all) identified 43.8% and 48.0% (*vs.* 49.7%) of future cases, respectively, while labeling as positive slightly smaller percentages (13.1% and 14.1% *vs.* 14.7%). Adding HbA1c and lipids improved future case detection slightly (48.7% and 53.1%).

**Table 2:** Diagnostic properties of continuous variable, one-step, and two-step screening strategies based on a clinical score and selected laboratory tests, as developed and validated in ELSA-Brasil.

Continuous variable strategies	Only clinical strategy			+FPG			+FPG, HbA1c, lipids		
	High risk	Sens	PPV	High risk	Sens	PPV	High risk	Sens	PPV
**≥20% probability of developing diabetes**	% (95%CI)	% (95%CI)	% (95%CI)	% (95%CI)	% (95%CI)	% (95%CI)	% (95%CI)	% (95%CI)	% (95%CI)
Test all	10.9 (10.0; 11.8)	31.6 (27.2; 35.9)	26.2 (22.4; 29.9)	14.7 (13.7; 15.7)	49.7 (44.9; 54.4)	30.6 (27.2; 34.0)	15.3 (14.3; 16.3)	54.5 (49.8; 59.2)	32.3 (28.9; 35.7)
First clinical score^a^, then combined with lab test(s) for									
67% of the sample at highest clinical risk				14.1 (13.1; 15.1)	48.0 (43.3; 52.7)	30.8 (27.3; 34.2)	14.7 (13.7; 15.7)	53.1 (48.4; 57.8)	32.8 (29.3; 36.2)
50% of the sample at highest clinical risk				13.1 (12.1; 14.0)	43.4 (38.7; 48.1)	30.1 (26.5; 33.7)	13.5 (12.5; 14.5)	48.7 (44.0; 53.4)	32.7 (29.0; 36.3)
33% of the sample at highest clinical risk				11.0 (10.1; 11.9)	35.0 (30.5; 39.6)	28.9 (25.0; 32.8)	11.3 (10.4; 12.2)	38.3 (33.7; 42.9)	30.7 (26.8; 34.6)
**≥15% probability of developing diabetes**									
Test all	20.1 (18.9; 21.2)	45.9 (41.2; 50.6)	20.7 (18.1; 23.3)	20.3 (19.2; 21.5)	64.0 (59.5; 68.6)	28.5 (25.6; 31.3)	20.7 (19.6; 21.9)	65.0 (60.5; 69.5)	28.4 (25.6; 31.2)
First clinical score^a^, then combined with lab test(s) for									
67% of the sample at highest clinical risk				19.5 (18.4; 20.6)	61.7 (57.1; 66.3)	28.6 (25.7; 31.5)	19.7 (18.6; 20.9)	63.1 (58.6; 67.7)	29.0 (26.1; 31.9)
50% of the sample at highest clinical risk				17.6 (16.6; 18.7)	56.6 (51.9; 61.3)	29.1 (26.0; 32.1)	17.9 (16.8; 19.0)	58.0 (53.4; 62.7)	29.3 (26.3; 32.4)
33% of the sample at highest clinical risk				14.6 (13.6; 15.6)	45.2 (40.5; 49.9)	28.1 (24.8; 31.5)	14.5 (13.6; 15.6)	46.2 (41.5; 50.9)	28.7 (25.3; 32.1)

Training sample N = 4,763. Validation sample, N = 4,762.

FPG, fasting plasma glucose; HbA1c, glycated hemoglobin; PPV, positive predictive value.

a A probability of developing diabetes over 7.4 years using just the clinical score of ≥6.5% initially selects 67% of the sample, one of ≥8.9% selects 50% of the sample, and one of ≥12% selects 33% of the sample.

When we lowered the score positivity cutoff to a 15% probability of future diabetes, rules identified considerably more future cases at the cost of labeling more of the sample as high risk. The clinical score alone performed better at this lower cutoff, labeling 20.1% at high risk and identifying 45.9% of those developing future diabetes. Adding FPG to this screening approach labeled 20.3% at high risk and identified 64.0% of future cases, and adding HbA1c and lipids labeled 15.3% at high risk and identified 65.0% of future cases. Testing FPG in only the 50% or 67% at highest clinical risk using the 15% cutoff identified slightly lower percentages (56.6% and 61.7% *vs.* 64.0%) of future cases, with marginally fewer labeled as positive (17.6% and 19.5% *vs.* 20.3%). Additionally adding HbA1c and lipids in these two-step approaches improved future case identification to 58.1% and 63.1%, respectively.

Two-step approaches, which advanced to laboratory testing only in the 33% at highest clinical risk while producing lower percentages (11.0% to 14.6%) of participants being labeled high risk, detected considerably fewer future cases (35.0% to 45.2%).

Compared with recommended one- or two-step categorical approaches presented in **Table 1**, continuous variable risk score strategies incorporating both clinical variables and glycemic testing labeled fewer individuals at high risk. As such, they created fewer false positives while still identifying a significant fraction of future cases. For example, when testing only the 67% at the highest clinical risk with FPG, HbA1c, and lipids, a 20% probability cutoff rule labeled only 14.7% as positive (*vs.* 47.2% for the ADA FPG and HbA1c lab-only strategy) and doubled the probability of developing diabetes among those at high risk (PPV = 32.8% *vs.* 15.7%) while detecting 53.1% of future cases. This strategy also compared favorably with the ADA two-step categorical approach, identifying more future cases (53.1% *vs.* 34.0%) while labeling only a slightly higher percentage as high risk (14.7% *vs.* 13.1%). Those labeled were more likely to develop diabetes (32.8% *vs.* 24.9%). Adding the 2hPG of an OGTT instead of an HbA1c as the additional laboratory test produced little benefit in strategies that included clinical variables and lipids (data not shown).

Of note, all of these continuous variable strategies presented PPVs, though higher than almost all of the categorical approaches, well below 50%, indicating the presence of many false positives. High false positivity was especially notable using a 15% cutoff or only the clinical score. False-positive rates (1 − PPVs) were usually ~5%–10% lower with the 20% probability cutoff.

**Supplementary Table 3** (for the 20% probability of developing diabetes cutoff) and **Supplementary Table 4** (for the 15% probability cutoff) present an expanded array of diagnostic properties for various one-step continuous variable strategies using laboratory testing, the clinical score, or both. As can be seen, the AUCs for continuous variable strategies were superior to those based only on categorical glycemic cutoffs, with the more elaborate continuous approaches achieving AUCs of ~0.85.

## 4 Discussion

We compared different screening strategies for diabetes in adults ≥ 35 years using easily obtainable clinical variables and laboratory results. Our findings showed that risk scores combining clinical variables and glycemic measures expressed continuously outperformed traditional laboratory-based categorical approaches and the two-step categorical approaches frequently recommended in national screening programs. Combining a continuously expressed FPG with clinical variables resulted in the largest gain in accuracy. Including additional laboratory results produced some further improvement. While all strategies identified considerably more false than true positives, those based on continuously expressed variables had a more balanced mix between greater future case detection and less false-positive labeling. Finally, using two-step strategies, with the first step evaluating only clinical variables to identify those initially at the highest risk and the second step adding laboratory testing only for those identified, considerably reduced the need for laboratory testing.

Three major national programs of diabetes prevention have defined specific rules to label high risk and initiate preventive intervention. In the United States, the CDC recommends lifestyle counseling for overweight individuals at high risk based on the ADA questionnaire, an ADA lab cutoff, or having a previous pregnancy complicated by gestational diabetes (22). The United Kingdom’s National Health Service recommends intervention for those positive with the two-step NICE screening strategy (12). The Finnish Diabetes Prevention Program (DPP) recommends using a score of ≥15 points on the FINDRISC questionnaire to identify high risk while also considering at high risk those with OGTT results above WHO cutoffs or a history of either coronary heart disease or previous gestational diabetes (5).

**Figure 3:**
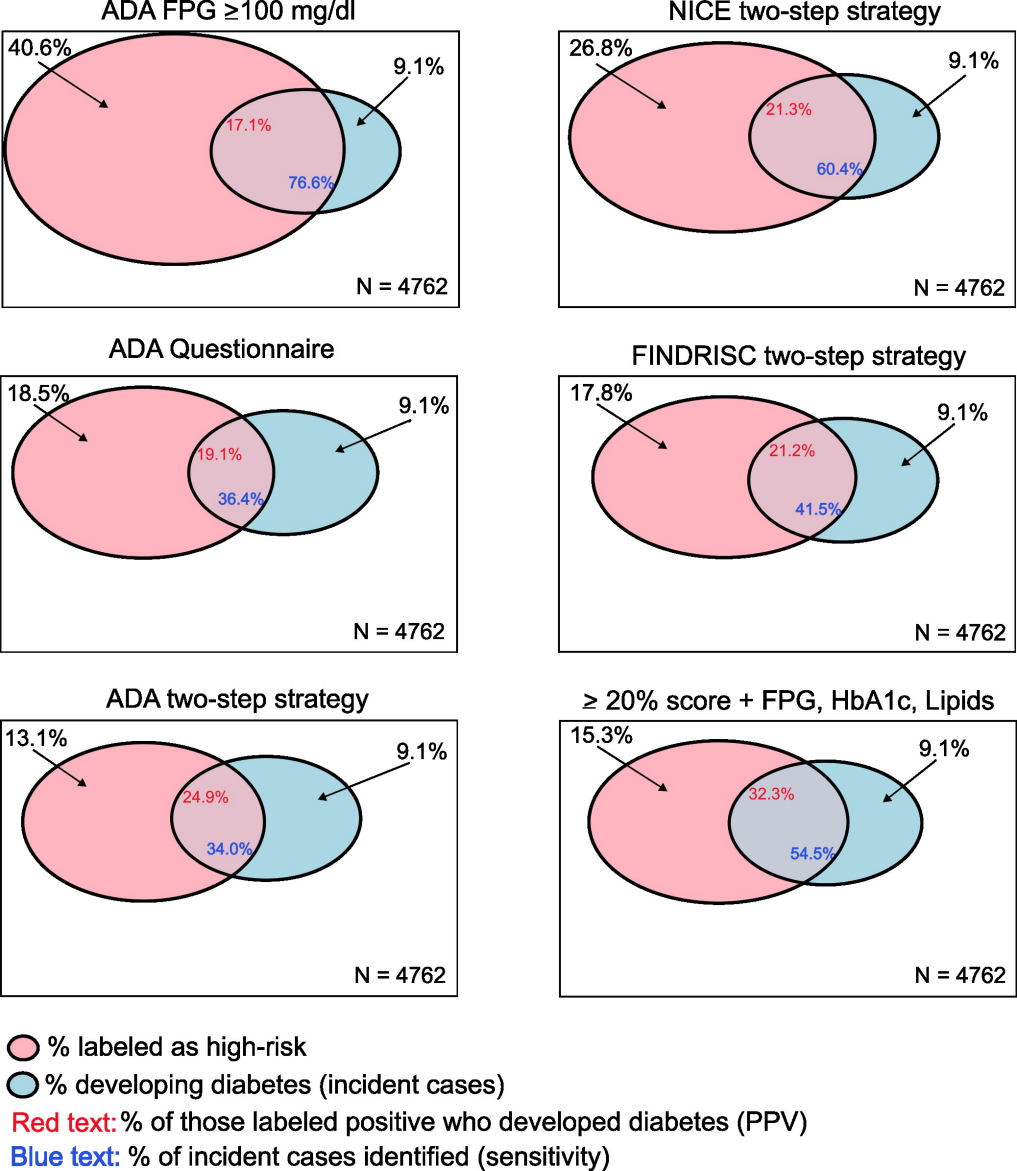
Graphical representation of three principal diagnostic metrics of different screening strategies. The box of each panel represents the whole sample. The red (left) circles represent the percentage of the sample who screened positive (labeled high risk) using each screening strategy, and the blue (right) circles represent the percentage of the sample who developed diabetes over follow-up. Positive predictive value (probability of those labeled as positive progressing to incident diabetes) is depicted by the percentage of the red circle intersecting with the blue circle (red number in the intersection), and screening test sensitivity (percentage of future incident cases identified) is depicted by the percentage of the blue circle within the red circle (blue number in the intersection).

Our findings indicate that the UK/NICE and especially the CDC/ADA approaches label a relatively high percentage of the sample as high risk, producing many referrals, a significant fraction of whom will not develop diabetes in the subsequent decade. Additionally, as shown in **Figure 3**, the approaches using ADA laboratory cutoffs, the ADA questionnaire, or both are internally inconsistent. Directly applied ADA glycemic testing labeled nearly 50% as high risk, but the ADA questionnaire labeled only 18% and the two-step approach only 13%. While the directly applied laboratory testing identified ~80% of cases, the questionnaire and two-step approaches identified little more than one-third of cases. The Finnish approach to screening, which sums those meeting other entry criteria with those presenting a high FINDRISC score, will likely also label a relatively high percentage as high risk and produce many false positives. Additionally, the two-step categorical strategies involving questionnaires and laboratory testing, as recommended by U.S. and U.K. authorities, were inferior to a similar continuous variable risk score as shown in the right panels of the figure. The continuous variable score labeled a smaller fraction of the population as at high risk than all but the ADA two-step strategy and produced a greater probability of those so labeled going on to develop diabetes than any of the other approaches.

As has been noted (24), almost all cost-effectiveness studies of screening followed by lifestyle interventions to prevent diabetes have been based on intensive interventions in non-community clinical trial settings. The effectiveness shown in these studies diminishes with their translation to community settings with less intensive interventions. With the use of the nationally recommended screening approaches, community programs also frequently recruit participants at lower risk of incident diabetes than those of the original clinical trials (2). This combination of a lower effectiveness of the intervention and a lower *a priori* probability of participants developing diabetes can markedly reduce the gains of screening. Lifestyle intervention in community settings, primarily in North America, has been estimated to prevent diabetes in only 3% of those enrolled (2). Improved titration of high-risk labeling through full use of clinical and laboratory information would improve the absolute probability of preventing diabetes through early detection. Hopefully, identifying additional influential risk factors from dietary, proteomic, DNA methylation, or metabolomic sources will permit further improvement of prediction. However, this gain may come at the cost of more sophisticated and thus more time-consuming and expensive strategies. Further, future advances in understanding the role of diet (25) and other factors in the pathogenesis of diabetes and in leading those with unhealthy habits to modify their human behavior will permit refinement in dietary and other lifestyle interventions.

The use of continuous variable screening strategies, here documented to be superior, as additionally found by another recent study (26), combined with patient preferences, seems more consistent with the current vision of precision and personalized medicine. To that end, **Table 3** presents practical, clinically relevant implications derived from our study. Frequent rescreening should mitigate the concern that the continuous variable rules shown here will detect fewer future cases than current ADA one-step approaches. The ADA recommends testing adults over 35 or presenting risk factors every 3 years and those with previously detected pre-diabetes every year (3).

**Table 3:** Main clinically relevant implications from findings based on diagnostic properties of screening strategies in the ELSA-Brasil validation sample.

• Combining sociodemographic and clinical factors with laboratory tests produces the best screening rules.
• Algorithms based on continuously expressed variables, made available *via* digital calculators, produce a better balance between detecting more future cases and creating fewer false positives.
• Fasting glucose is best among available laboratory tests considering feasibility, accuracy, and cost issues.
• When feasible, including glycated hemoglobin, HDL-c, and triglycerides will provide additional prediction in screening.
• Two-step strategies, first identifying those at higher risk based only on readily available clinical information and then testing them for hyperglycemia, can reduce laboratory testing with minimal loss in detecting future cases.

HDL-c, high-density lipoprotein cholesterol.

A potential limitation to our study is that our rules using HbA1c performed relatively poorly, suggesting that greater laboratory error in HbA1c determination, although not reflected in our evaluation of its reliability coefficient (27), could have been present. Such imprecision could have led to underestimating the benefit of including HbA1c in strategies. Additionally, as in all evaluations of clinical predictive rules, our results are based on our sample’s pretest probability—the probability of developing diabetes over a 7.5-year follow-up. Settings with considerably greater or lesser incidence would need to calibrate our rules to their population’s risk of developing diabetes. However, as shown by the Global Burden of Disease Study, the incidence of type 2 diabetes is not greatly different in Brazil than in most other parts of the world (**Supplementary Figure 1**).

Our study presents several strengths. Its outcome of future incident diabetes and its focus on the most relevant screening metrics permit clinically relevant, head-to-head comparisons of different strategies. Our ascertainment of diabetes required confirmation, approximating it to the clinical definition, giving our findings greater generalizability. Standardized collection of data and centralized laboratory measurement add quality and precision to our results. Our relatively large sample size allows for more precise estimates of diagnostic properties. Rates of obesity and central obesity and other diabetes risk factors in ELSA-Brasil, being a contemporary cohort, are more in line with their current prevalence, permitting more easily generalizable clinical scores. Finally, our provision of an online calculator permits immediate use of findings.

## 5 Conclusions

All evaluated screening strategies to predict future diabetes are far from perfect. However, risk scores combining clinical variables with glycemic measures expressed in a continuous form are superior to traditional screening strategies and currently recommended two-step categorical strategies. National programs and those making recommendations should favor continuous variable scores in their recommended screening strategies to maximize the potential benefit for those invited to screening programs while guaranteeing an adequate balance between benefits and costs. They should also make explicit the impact of their screening strategy recommendations in terms of the percentage labeled as high risk and the probability that those so labeled, without intervention, will develop diabetes in the foreseeable future.

## Data availability statement

The raw data supporting the conclusions of this article will be made available by the authors, without undue reservation.

## Ethics statement

The studies involving human participants were reviewed and approved by Comitê de Ética em Pesquisa do Hospital de Clínicas de Porto Alegre. The patients/participants provided their written informed consent to participate in this study.

## Author contributions

Concept and design: PB, MS, and BD. Acquisition, analysis, or interpretation of data: PB, MS, AV, JM, PV, SB, and BD. Drafting of the manuscript: PB and BD. All authors made critical revisions to the manuscript for important intellectual content. All authors contributed to the article and approved the submitted version.

## References

[1] Jonas DE Crotty K Yun JDY Middleton JC Feltner C Taylor-Phillips S. Screening for prediabetes and type 2 diabetes: updated evidence report and systematic review for the US preventive services task force. JAMA (2021) 326(8):744. doi: 10.1001/jama.2021.10403 34427595

[2] Galaviz KI Weber MB Straus A Haw JS Narayan KMV Ali MK. Global diabetes prevention interventions: a systematic review and network meta-analysis of the real-world impact on incidence, weight, and glucose. Diabetes Care (2018) 41(7):1526–34. doi: 10.2337/dc17-2222 PMC6463613 29934481

[3] American Diabetes Association. 2. classification and diagnosis of diabetes: *Standards of medical care in diabetes–2018* . Diabetes Care (2018) 41(Supplement 1):S13–27. doi: 10.2337/dc18-S002 29222373

[4] Preventive Services Task Force US Davidson KW Barry MJ Mangione CM Cabana M Caughey AB. Screening for prediabetes and type 2 diabetes: US preventive services task force recommendation statement. JAMA (2021) 326(8):736. doi: 10.1001/jama.2021.12531 34427594

[5] Saaristo T Peltonen M Keinänen-Kiukaanniemi S Vanhala M Saltevo J Niskanen L. National type 2 diabetes prevention programme in Finland: FIN-D2D. Int J Circumpolar Health (2007) 66(2):101–12. doi: 10.3402/ijch.v66i2.18239 17515250

[6] Albright AL Gregg EW. Preventing type 2 diabetes in communities across the U.S. Am J Prev Med (2013) 44(4):S346–51. doi: 10.1016/j.amepre.2012.12.009 PMC4539613 23498297

[7] Torjesen I. NHS England Rolls out world’s first national diabetes prevention programme. BMJ (2016) 21:i1669. doi: 10.1136/bmj.i1669 27006393

[8] World Health Organization International Diabetes Federation. Definition and diagnosis of diabetes mellitus and intermediate hyperglycaemia: report of a WHO/IDF consultation (2006). Available at: http://www.who.int/diabetes/publications/diagnosis_diabetes2006/en/.

[9] Gillett MJ. International expert committee report on the role of the A1c assay in the diagnosis of diabetes: diabetes care. Clin Biochem Rev (2009) 30(4):197–200. 20011212 PMC2791773

[10] Take the test - prediabetes | diabetes | CDC (2022). Available at: https://www.cdc.gov/PREDIABETES/RISKTEST/.

[11] Diabetes UK – know your risk of type 2 diabetes (2022). Available at: https://riskscore.diabetes.org.uk/start.

[12] NICE. Type 2 diabetes: prevention in people at high risk (2012). Available at: https://www.nice.org.uk/guidance/ph38/resources/type-2-diabetes-prevention-in-people-at-high-risk-pdf-1996304192197.

[13] Hippisley-Cox J Coupland C. Development and validation of QDiabetes-2018 risk prediction algorithm to estimate future risk of type 2 diabetes: cohort study. BMJ (2017) 20:j5019. doi: 10.1136/bmj.j5019 PMC5694979 29158232

[14] Noble D Mathur R Dent T Meads C Greenhalgh T. Risk models and scores for type 2 diabetes: systematic review. BMJ (2011) 343(nov28 1):d7163–3. doi: 10.1136/bmj.d7163 PMC3225074 22123912

[15] Lucaroni F Cicciarella Modica D Macino M Palombi L Abbondanzieri A Agosti G. Can risk be predicted? an umbrella systematic review of current risk prediction models for cardiovascular diseases, diabetes and hypertension. BMJ Open (2019) 9(12):e030234. doi: 10.1136/bmjopen-2019-030234 PMC6937066 31862737

[16] Ayensa-Vazquez JA Leiva A Tauler P López-González AA Aguiló A Tomás-Salvá M. Agreement between type 2 diabetes risk scales in a Caucasian population: a systematic review and report. JCM (2020) 9(5):1546. doi: 10.3390/jcm9051546 32443837 PMC7290893

[17] Schmidt MI Duncan BB Mill JG Lotufo PA Chor D Barreto SM. Cohort profile: longitudinal study of adult health (ELSA-brasil). Int J Epidemiol (2015) 44(1):68–75. doi: 10.1093/ije/dyu027 24585730 PMC4339754

[18] Aquino EML Barreto SM Bensenor IM Carvalho MS Chor D Duncan BB. Brazilian Longitudinal study of adult health (ELSA-brasil): objectives and design. Am J Epidemiol (2012) 175(4):315–24. doi: 10.1093/aje/kwr294 22234482

[19] Forechi L Mill JG Griep RH Santos I Pitanga F Molina M del CB. Adherence to physical activity in adults with chronic diseases: ELSA-brasil. Rev saúde pública (2018) 52:31. doi: 10.11606/S1518-8787.2018052000215 29641656 PMC5893266

[20] Molina M del CB Benseñor IM Cardoso L de O Velasquez-Melendez G Drehmer M Pereira TSS. Reprodutibilidade e validade relativa do questionário de frequência alimentar do ELSA-brasil. Cad Saúde Pública (2013) 29(2):379–89. doi: 10.1590/S0102-311X2013000200024 23459823

[21] Expert Committee on the Diagnosis and Classification of Diabetes Mellitus. Report of the expert committee on the diagnosis and classification of diabetes mellitus. Diabetes Care (2003) 26(Supplement 1):S5–20. doi: 10.2337/diacare.26.2007.s5 12502614

[22] Expert Committee on the Diagnosis and Classification of Diabetes Mellitus. Lifestyle change program details | national diabetes prevention program | CDC (2022). Available at: https://www.cdc.gov/diabetes/prevention/lcp-details.html.

[23] Lindström J Tuomilehto J. The diabetes risk score: apractical tool to predict type 2 diabetes risk. DiabetesCare (2003) 26:725–731. doi: 10.2337/diacare.26.3.725 12610029

[24] Roberts S Barry E Craig D Airoldi M Bevan G Greenhalgh T. Preventing type 2 diabetes: systematic review of studies of cost-effectiveness of lifestyle programmes and metformin, with and without screening, for pre-diabetes. BMJ Open (2017) 7(11):e017184. doi: 10.1136/bmjopen-2017-017184 PMC5695352 29146638

[25] Neuenschwander M Ballon A Weber KS Norat T Aune D Schwingshackl L. Role of diet in type 2 diabetes incidence: umbrella review of meta-analyses of prospective observational studies. BMJ (2019) 366:l2368:1–9. doi: 10.1136/bmj.l2368 PMC6607211 31270064

[26] Wilkinson L Yi N Mehta T Judd S Garvey WT. Development and validation of a model for predicting incident type 2 diabetes using quantitative clinical data and a Bayesian logistic model: a nationwide cohort and modeling study. PloS Med (2020) 17(8):e1003232. doi: 10.1371/journal.pmed.1003232 32764746 PMC7413417

[27] Ladwig R Vigo A Fedeli LMG Chambless LE Bensenor I Schmidt MI. Variability in baseline laboratory measurements of the Brazilian longitudinal study of adult health (ELSA-brasil). Braz J Med Biol Res (2016) 49(9):1–19. http://www.scielo.br/scielo.php?script=sci_arttext&pid=S0100-879X2016000900701&tlng=en. doi: 10.1590/1414-431x20165381 PMC4988480 27533768

