# Supplementary material for: Optimizing strategies to identify high risk of developing type 2 diabetes
Source: Front Endocrinol (Lausanne). 2023 Jun 28;14:1166147. doi: 10.3389/fendo.2023.1166147 (PMC10338007; doi:10.3389/fendo.2023.1166147)

Supplementary Material

**Optimizing Strategies to Identify High Risk of Developing Type 2 Diabetes**

Paula Andreghetto Bracco^*^, Maria Inês Schmidt, Alvaro Vigo, José Geraldo Mill, Pedro Guatimosim Vidigal, Sandhi Maria Barreto, Mária de Fátima Sander, Maria de Jesus Mendes da Fonseca, Bruce Bartholow Duncan^3^

*** Correspondence:** Dr. Paula A. Bracco: [paula.abracco@gmail.com](mailto:paula.abracco@gmail.com)

Formulas derived through logistic regression to estimate the probability of developing diabetes for each of our risk models (ELSA-Brasil training dataset, N=4763):

1. **Only FPG**

*x* = - **14.8357** + **0.1227***FPG

1. **Only HbA1c**

*x* = - **9.9902** + **1.4518***HbA1c

1. **FPG + HbA1c**

*x* = - **19.3082** + **0.1117***FPG + **1.0570***HbA1c

1. **FPG + HbA1c + Lipids**

*x* – **17.8750** + **0.1042***FPG + **1.0870***HbA1c +**0.00188***triglycerides - **0.0207***HDL-c

1. **FPG + 2hPG + HbA1c**

*x* = - **19.1844** + **0.0844***FPG + **0.0253***2hPG + **0.9508***HbA1c

1. **FPG + 2hPG + HbA1c + Lipids**

*x* = - **17.9944** + **0.0810***FPG + **0.0242***2hPG + **0.9739***HbA1c + **0.000822***triglycerides -**0.0181***HDL-c

1. **Clinical variables:**

*x* = - **8.2347** - **0.00213***woman - **0.1736***white + **0.3080***parental history of diabetes + **0.1711***hypertension + **0.0219***age + **0.0768***body mass index + **0.0306***waist circumference

1. **Clinical + FPG:**

*x* = - **17.6048** + **0.1801***woman - **0.1886***white + **0.2695***parental history of diabetes + **0.0530***hypertension + **0.00110***age + **0.0821***body mass index + **0.0159***waist circumference + **0.1134***FPG

1. **Clinical + HbA1c:**

*x* = - **13.5399** - **0.0412***women -**0 .1206***white + **0.3136***parental history of diabetes + **0.1597***hypertension + **0.0107***age + **0.0712***body mass index + **0.0274***waist circumference + **1.1958***HbA1c

1. **Clinical + FPG, HbA1c**

*x* = - **20.8726** + **0.1232***women -**0.1486***white + **0.2814***parental history of diabetes + **0.0478***hypertension - **0.00564***age + **0.0781***body mass index + **0.0141***waist circumference + **0.1047***FPG + **0.9041***HbA1c

1. **Clinical + FPG, 2hPG**

*x* = - **17.3950** + **0.1873***women - **0.1794***white + **0.2475***parental history of diabetes + **0.0169***hypertension - **0.00924***age + **0.0763***body mass index + **0.0111***waist circumference + **0.0925***FPG + **0.0237***2hPG

1. **Clinical + FPG, 2hPG, Lipids**

x = - **16.2713** + **0.2695***women -**0.1914***white + **0.2436***parental history of diabetes + **0.0140***hypertension - **0.00480***age + **0.0757***body mass index + **0.00774***waist circumference + **0.0916***FPG + **0.0227***2hPG + **0.000921***triglycerides - **0.0180***HDL-c

1. **Clinical + FPG, HbA1c, Lipids**

*x* = - **19.5067** + **0.2330***women - **0.1570***white + **0.2810***parental history of diabetes + **0.0322***hypertension + **0.000088***age + **0.0797***body mass index + **0.00854***waist circumference + **0.1013***FPG + **0.9031***HbA1c +**0.00193***triglycerides - **0.0214***HDL-c

**Note:**

FPG=fasting plasma glucose; 2hPG=2h plasma glucose; HbA1c=glycated hemoglobin; HDL-c: high density lipoprotein cholesterol;

FPG, HDL-c, and triglycerides are in *mg/dl*; HbA1c in percent (*%*), BMI in *kg/m2*, waist circumference in *cm*, age in *years*. All other variables take a value of 1 when the condition is present.

# Supplementary Tables

**Supplementary Table 1.** Baseline characteristics of our total, training, and validation datasets, presented in either frequency (categorical variables) or unadjusted means (continuous variables) and respective confidence intervals. ELSA-Brasil validation sample, N=4762.

|  | Training Set n=4763 | | Validation Set n=4762 | |
| --- | --- | --- | --- | --- |
|  | New Cases of Diabetes | | New Cases of Diabetes | |
| Baseline Characteristics | Yes n=433 (9.1%) | No n=4330 | Yes n=431 (9.1%) | No n=4331 |
| Age (Years) | 52.1  (51.3-52.9) | 50.1  (49.8-50.4) | 52.3  (51.5-53.0) | 50.5  (50.3-50.8) |
| Women | 232  (53.6%; 48.9-58.3) | 2478  (57.2%; 55.8-58.7) | 216  (50.1%; 45.4-54.8) | 2511  (58.0%; 56.5-59.5) |
| Self-reported ethnicity |  |  |  |  |
| White | 197  (45.5%; 40.8-50.2) | 2380  (55.0%; 53.5-56.5) | 210  (48.7%; 44.0-53.4) | 2360  (54.5%; 53.0-56.0) |
| Educational attainment |  |  |  |  |
| Incomplete elementary | 24  (5.5%; 3.4-7.7) | 150  (3.5%; 2.9-4.0) | 30  (7.0%; 4.6-9.3) | 150  (3.5%; 2.9-4.0) |
| Incomplete secondary | 43  (9.9%; 7.2-12.8) | 205  (4.7%; 4.1-5.4) | 43  (10.0%; 7.2-12.8) | 219  (5.1%; 4.4-5.7) |
| Secondary school | 173  (40.0%; 35.3-44.6) | 1488  (34.4%; 33.0-35.8) | 161  (37.3%; 32.8-41.9) | 1435  (33.1%; 31.7-34.5) |
| University degree | 193  (44.6%; 39.9-49.3) | 2487  (57.4%; 56.0-58.9) | 197  (45.7%; 41.0-50.4) | 2527  (58.4%; 56.9-59.8) |
| Smoking |  |  |  |  |
| Never | 220  (50.8%; 46.1-55.5) | 2760  (62.5%; 61.1-63.9) | 229  (53.1%; 48.4-57.8) | 2615  (60.4%; 58.9-61.8) |
| Former | 150  (34.6%; 30.2-39.1) | 1150  (26.6%; 25.2-27.9) | 128  (29.7%; 25.4-43.0) | 1179  (27.2%; 25.9-28.5) |
| Current | 63  (14.6%; 11.2-17.9) | 474  (11.0%; 10.0-11.9) | 74  (17.7%; 13.6-20.7) | 537  (12.4%; 11.4-13.4) |
| Daily fruit or vegetable consumption | 345  (79.7%; 75.9-83.5) | 3418  (78.9%; 77.7-80.2) | 334  (77.5%; 73.6-81.4) | 3443  (79.5%; 78.3-80.7) |
| Leisure physical activity  (MET-minutes/week) | 564.1  (459.4-668.8) | 701.9  (668.6-735.3) | 570.2  (485.0-655.5) | 721.1  (686.8-755.3) |
| Parental history of diabetes | 173  (40.0; 35.3-44.6) | 1144  (26.4%; 25.1-27.7) | 178  (41.3%; 36.7-46.0) | 1147  (26.5%; 25.2-27.8) |
| Hypertension | 194  (44.8%; 40.1-49.5) | 1112  (25.7%; 24.4-27.0) | 195  (45.2%; 40.5-49.9) | 1130  (26.1%; 24.8-27.4) |
| BMI (kg/m^2^) | 29.6  (29.2-30.1) | 26.1  (25.9-26.2) | 29.4  (29.0-29.9) | 26.0  (25.9 - 26.2) |
| Waist circumference (cm) | 97.9  (96.5-98.7) | 88.3  (87.9-88.6) | 97.1  (96.1-98.2) | 88.1  (87.7 - 88.4) |

**Supplementary Table 2.** Diagnostic properties of laboratory-based categorical screening strategies to detect future diabetes, assuming a one-step, categorical cutoff screening strategy. ELSA-Brasil validation sample, N=4762.

|  | **AUC** | **High-risk** | **Sensitivity** | **Specificity** | **PPV** | **NPV** | **NRI** | **NRIp** |
| --- | --- | --- | --- | --- | --- | --- | --- | --- |
| **Laboratory Testing Strategies** | **(95%CI)** | **%**  **(95%CI)** | **%**  **(95%CI)** | **%**  **(95%CI)** | **%**  **(95%CI)** | **%**  **(95%CI)** | **%** |  |
| Only FPG ≥100 mg/dl (ADA) | 0.698  (0.676;0.719) | 40.6 (39.3;42.0) | 76.6 (72.6;80.6) | 62.9 (61.5;64.4) | 17.1 (15.4;18.7) | 96.4 (95.7;97.1) | **Reference** |  |
| Only FPG ≥110 mg/dl (WHO) | 0.660  (0.637;0.684) | 11.0 (10.1;11.9) | 40.1  (35.5;44.8) | 91.9  (91.1;92.7) | 33.0 (29.0;37.0) | 93.9 (93.2;94.6) | -7.4 | 0.002 |
| Only 2hPG ≥140 mg/dl | 0.710  (0.686;0.734) | 18.9  (17.8;20.0) | 57.2  (52.5;61.9) | 84.9  (83.8;86.0) | 27.3 (24.4;30.3) | 95.2  (94.5;95.9) | 2.3 | 0.45 |
| Only HbA1c ≥5.7% (39mmol/mol) (ADA) | 0.576  (0.554;0.598) | 14.2  (13.2;15.1) | 28.0  (23.7;32.2) | 87.2  (86.2;88.2) | 17.8 (!4.9;20.7) | 92.4  (91.6;93.2) | -24.3 | <0.001 |
| Only HbA1c ≥ 6.0% (42mmol/mol) (IEC) | 0.528  (0.513;0.542) | 5.0  (4.4;5.7) | 10.0 (7.2;12.9) | 95.5  (94.8;96.1) | 18.0  (13.1;22.9) | 91.5 (90.6;92.3) | -34.3 | <0.001 |
|  |  |  |  |  |  |  |  |  |
| FPG >100 mg/dl or 2hPG ≥140 mg/dl (ADA) | 0.715  (0.697;0.733) | 47.5  (46.1;49.0) | 86.7  (83.5;89.9) | 56.4  (54.9;57.8) | 16.5  (15.0;18.1) | 97.7  (97.1;98.3) | 3.4 | 0.022 |
| FPG >110 mg/dl or 2hPG ≥140 mg/dl (WHO) | 0.749  (0.727;0.772) | 24.9  (23.6;26.1) | 70.2  (65.8;74.5) | 79.7  (78.4;80.9) | 25.6  (23.1;28.1) | 96.4  (95.8;97.0) | 10.1 | <0.001 |
| FPG >100 mg/dl or HbA1c ≥ 5.7% (39mmol/mol) (ADA) | 0.693  (0.673;0.712) | 47.2  (45.8;48.6) | 82.3  (78.7;85.9) | 56.3  (54.8;57.8) | 15.7  (14.2;17.2) | 97.0 (96.2;97.6) | -1.2 | 0.28 |
| FPG >110 mg/dl or HbA1c ≥ 6.0% (42mmol/mol) (IEC) | 0.664  (0.640;0.688) | 15.0  (14.0;16.0) | 44.8  (40.0;49.5) | 88.0  (87.0;88.9) | 27.0  (23.7;30.2) | 94.1  (93.4;94.9) | -7.1 | 0.006 |
|  |  |  |  |  |  |  |  |  |
| FPG >100 mg/dl, 2hPG ≥140 mg/dl or HbA1c ≥5.7% (39mmol/mol) (ADA) | 0.699  (0.683;0.716) | 53.0  (51.5;54.4) | 89.2  (86.3;92.1) | 50.7  (49.1;52.2) | 15.2  (13.8;16.7) | 97.9 (97.3;98.5) | -0.2 | 0.9 |
| FPG >110 mg/dl, 2hPG ≥140 mg/dl or HbA1c ≥ 6.0% (42mmol/mol) (WHO/IEC) | 0.750  (0.728;0.772) | 27.8  (26.5;29.1) | 73.4  (68.9;77.5) | 76.7  (75.4;78.0) | 23.8  (21.5;26.2) | 96.7  (96.0;97.3) | 10.0 | <0.001 |

AUC=area under the ROC curve; PPV=positive predictive value; NPV=negative predictive value; NRI=net-reclassification index; NRIp=p value for net reclassication index; FPG=fasting plasma glucose; 2hPG=2h plasma glucose; HbA1c=glycated hemoglobina; ADA=American Diabetes Association; WHO=World Health Organization; IEC=International Expert Committee

**Supplementary Table 3.** Diagnostic properties of different screening strategies based on continuous variables to detect future diabetes when a positive test is defined as identifying risk of incident diabetes ≥20%. ELSA-Brasil validation sample, N=4762.

|  | **AUC** | **High Risk** | **Sens** | **Spec** | **PPV** | **NPV** | **NRI**^†^ | **NRIp** |
| --- | --- | --- | --- | --- | --- | --- | --- | --- |
| **Strategy** | (95%CI) | % (95%CI) | % (95%CI) | % (95%CI) | % (95%CI) | % (95%CI) | % |  |
| Only Lab |  |  |  |  |  |  |  |  |
| Only FPG | 0.776 (0.753;0.799) | 11.0 (10.1;11.9) | 40.1 (35.5;44.8) | 91.9 (91.1;92.7) | 33.0 (29.0;37.0) | 93.9 (93.2;94.6) | -7.4 | 0.002 |
| Only HbA1c | 0.668 (0.641;0.695) | 5.0 (4.4;5.6) | 10.0 (7.2;12.9) | 95.5 (94.9;96.1) | 18.0 (13.1;22.9) | 91.5 (90.6;92.3) | -3.3 | < 0.001 |
| FPG + 2hPG | 0.830 (0.809;0.850) | 11.2 (10.3;12.0) | 49.2 (44.4;53.9) | 92.5 (91.7;93.3) | 39.4 (35.2;43.5) | 94.8 (94.1;95.5) | 1.8 | 0.45 |
| FPG + HbA1c | 0.793 (0.772;0.815) | 10.8 (9.9;11.7) | 41.5 (36.8;46.2) | 92.3 (91.5;93.1) | 34.7 (30.6;38.8) | 94.1 (93.4;94.8) | -6.1 | 0.012 |
| FPG + HbA1c + Lipids | 0.813 (0.792;0.834) | 11.3 (10.4;12.2) | 44.4 (39.6;49.2) | 91.9 (91.1;92.7) | 35.2 (31.2;39.2) | 94.4 (93.7;95.1) | -3.5 | 0.15 |
| FPG + 2hPG + HbA1c | 0.839 (0.820;0.859) | 11.6 (10.6;12.5) | 49.9 (45.1;54.7) | 92.1 (91.3;92.9) | 38.4 (34.3;42.4) | 94.9 (94.2;95.6) | 1.8 | 0.47 |
| FPG + 2hPG + HbA1c + Lipids | 0.849 (0.830;0.868) | 11.6 (10.7;12.5) | 50.7 (45.9;55.5) | 92.0 (91.1;92.8) | 38.6 (34.6;42.7) | 95.0 (94.3;95.6) | 26.5 | 0.28 |
|  |  |  |  |  |  |  |  |  |
| Only clinical score | 0.750 (0.728;0.772) | 10.9 (10.0;11.8) | 31.6 (27.2;35.9) | 91.1 (90.3;92.0) | 26.2 (22.4;29.9) | 93.1 (92.3;93.8) | -16.8 | < 0.001 |
|  |  |  |  |  |  |  |  |  |
| Clinical score + 1 Lab Test |  |  |  |  |  |  |  |  |
| Clinical + FPG | 0.825 (0.805;0.844) | 14.7 (13.7;15.7) | 49.7 (44.9;54.4) | 88.8 (87.9;89.7) | 30.6 (27.2;34.0) | 94.7 (94.0;95.4) | -1.0 | 0.65 |
| Clinical + HbA1c | 0.774 (0.753;0.796) | 12.3 (11.4.13.3) | 35.0 (30.5;39.5) | 89.9 (89.0;90.1) | 25.6 (22.0;29.1) | 93.3 (92.6;94.1) | -15.0 | < 0.001 |
|  |  |  |  |  |  |  |  |  |
| Clinical score + >1 Lab Test |  |  |  |  |  |  |  |  |
| Clinical + FPG, HbA1c | 0.835 (0.816;0.853) | 13.9 (12.9;14.9) | 48.3 (43.5;53.0) | 89.5 (88.5;90.4) | 31.2 (27.7;34.8) | 94.6 (93.9;95.3) | -2.1 | 0.38 |
| Clinical + FPG, HbA1c, Lipids | 0.846 (0.828;0.864) | 15.3 (14.3;16.3) | 54.9 (50.2;59.6) | 88.6 (87.7;89.6) | 32.3 (28.9;35.7) | 95.2 (94.6;95.9) | 3.8 | 0.11 |
| Clinical + FPG, 2hPG | 0.854 (0.835;0.872) | 14.1 (13.1;15.1) | 57.7 (53.0;62.3) | 90.0 (89.1;90.9) | 36.5 (32.9;40.2) | 95.5 (94.9;96.2) | 7.9 | 0.001 |
| Clinical + FPG, 2hPG, Lipids | 0.862 (0.844;0.879) | 14.6 (13.6;15.6) | 59.9 (55.2;64.6) | 89.7 (88.8;90.6) | 36.7 (33.1;40.2) | 95.8 (95.1;96.4) | 9.9 | < 0.001 |

^†^FPG >100 mg/dl is the reference for NRI calculations;

Sens=sensitivity; Spec=specificity; PPV=positive predictive value; NPV=negative predictive value; NRI = net-reclassification index; NRIp = NRI p-value; FPG=fasting plasma glucose; 2hPG=2h plasma glucose; HbA1c=glycated hemoglobin Lipids=triglycerides and high density lipoprotein cholesterol.

**Supplementary Table 4.** Diagnostic properties of different one-step screening strategies based on continuous variables to detect future diabetes when a positive test is defined as identifying risk of incident diabetes ≥15%. ELSA-Brasil validation sample, N=4762.

|  | **AUC** | **High-Risk** | **Sens** | **Spec** | **PPV** | **NPV** | **NRI**^†^ | **NRIp** |
| --- | --- | --- | --- | --- | --- | --- | --- | --- |
| **Strategy** | (95%CI) | % (95%CI) | % (95%CI) | % (95%CI) | % (95%CI) | % (95%CI) | % |  |
| Only Lab |  |  |  |  |  |  |  |  |
| Only FPG | 0.776 (0.753;0.799) | 17.1 (16.0;18.2) | 51.7 (47.0;56.5) | 86.3 (85.3;87.4) | 27.4 (24.3;30.4) | 94.7 (94.0;95.4) | -1.4 | 0.51 |
| Only HbA1c | 0.668 (0.641;0.695) | 14.1 (13.1;15.1) | 28.0 (23.7;32.2) | 87.2 (86.2;88.2) | 17.8 (14.9;20.7) | 92.4 (91.6;93.2) | -24.6 | < 0.001 |
| FPG + 2hPG | 0.830 (0.809;0.850) | 15.7 (14.7;16.7) | 59.3 (54.6;64.0) | 88.4 (87.5;89.4) | 33.7 (30.3;37.1) | 95.6 (95.0;96.3) | 7.9 | < 0.001 |
| FPG + HbA1c | 0.793 (0.772;0.815) | 16.1 (15.0;17.1) | 50.8 (46.1;55.6) | 87.4 (86.4;88.4) | 28.5 (25.3;31.7) | 94.7 (94.0;95.4) | -1.7 | 0.46 |
| FPG + HbA1c + Lipids | 0.813 (0.792;0.834) | 16.9 (15.8;18.0) | 54.2 (49.5;58.9) | 86.8 (85.8;97.8) | 28.9 (25.7;32.0) | 95.0 (94.4;95.7) | 1.2 | 0.59 |
| FPG + 2hPG + HbA1c | 0.839 (0.820;0.859) | 16.1 (15.1;17.2) | 60.5 (55.9;65.2) | 88.0 (87.1;89.0) | 33.4 (30.0;36.7) | 95.8 (95.1;96.4) | 8.4 | < 0.001 |
| FPG + 2hPG + HbA1c + Lipids | 0.849 (0.830;0.868) | 16.4 (15.3;17.4) | 62.3 (57.7;67.0) | 87.9 (86.9;88.9) | 33.7(30.4;37.0) | 95.9 (95.3;96.6) | 10.2 | < 0.001 |
|  |  |  |  |  |  |  |  |  |
| Only clinical score | 0.750 (0.728;0.772) | 20.1 (18.9;21.2) | 45.9 (41.2;50.6) | 82.5 (81.4;83.6) | 20.7 (18.1;23.3) | 93.4 (93.1;94.6) | -11.1 | < 0.001 |
|  |  |  |  |  |  |  |  |  |
| Clinical score + 1 Lab Test |  |  |  |  |  |  |  |  |
| Clinical + FPG | 0.825 (0.805;0.844) | 20.4 (19.2;21.5) | 64.0 (59.5;68.6) | 84.0 (82.9;85.1) | 28.5 (25.6;31.3) | 95.9 (95.3;96.5) | 8.5 | < 0.001 |
| Clinical + HbA1c | 0.774 (0.753;0.796) | 20.5 (19.4;21.7) | 52.9 (48.2;57.6) | 82.6 (81.5;83.8) | 23.2 (20.6;25.9) | 94.7 (93.9;95.4) | -4.2 | 0.16 |
|  |  |  |  |  |  |  |  |  |
| Clinical score + >1 Lab Test |  |  |  |  |  |  |  |  |
| Clinical + FPG, HbA1c | 0.835 (0.816;0.853) | 19.8 (18.6;20.9) | 60.6 (56.0;65.2) | 84.3 (83.2;85.3) | 27.6 (24.8;30.5) | 95.6 (94.9;96.2) | 5.0 | 0.03 |
| Clinical + FPG, HbA1c, Lipids | 0.846 (0.828;0.864) | 20.7 (19.6;21.9) | 65.4 (60.9;69.9) | 83.7 (82.6;84.8) | 28.4 (25.6;31;2) | 96.1 (95.5;96.7) | 9.3 | < 0.001 |
| Clinical + FPG, 2hPG | 0.854 (0.835;0.872) | 19.2 (18.0;20.3) | 67.1 (62.6;71.5) | 85.3 (84.3;86.4) | 31.3 (28.2;34.3) | 96.3 (95.7;96.9) | 12.6 | < 0.001 |
| Clinical + FPG, 2hPG, Lipids | 0.862 (0.844;0.879) | 20.0 (18.8;21.1) | 69.8 (65.4;74.2) | 84.7 (83.6;85.8 | 31.2 (28.2;34.1) | 95.6 (96.0;97.2) | 14.8 | < 0.001 |

^†^FPG >100 mg/dl is the reference for NRI calculations;

Sens=sensitivity; Spec=specificity; PPV=positive predictive value; NPV=negative predictive value; NRI = net-reclassification index; NRIp = NRI p-value; FPG=fasting plasma glucose; 2hPG=2h plasma glucose; HbA1c=glycated hemoglobin Lipids=triglycerides and high density lipoprotein cholesterol.

**Supplementary Figure 1.** All-age incidence of type 2 diabetes mellitus in Brazil, in comparison to differing WHO regions of the world. Source: Institute for Health Metrics and Evaluation (IHME). GBD Compare. Seattle, WA: IHME, University of Washington, 2019. Available from http://vizhub.healthdata.org/gbd-compare. (Accessed April 19, 2023)


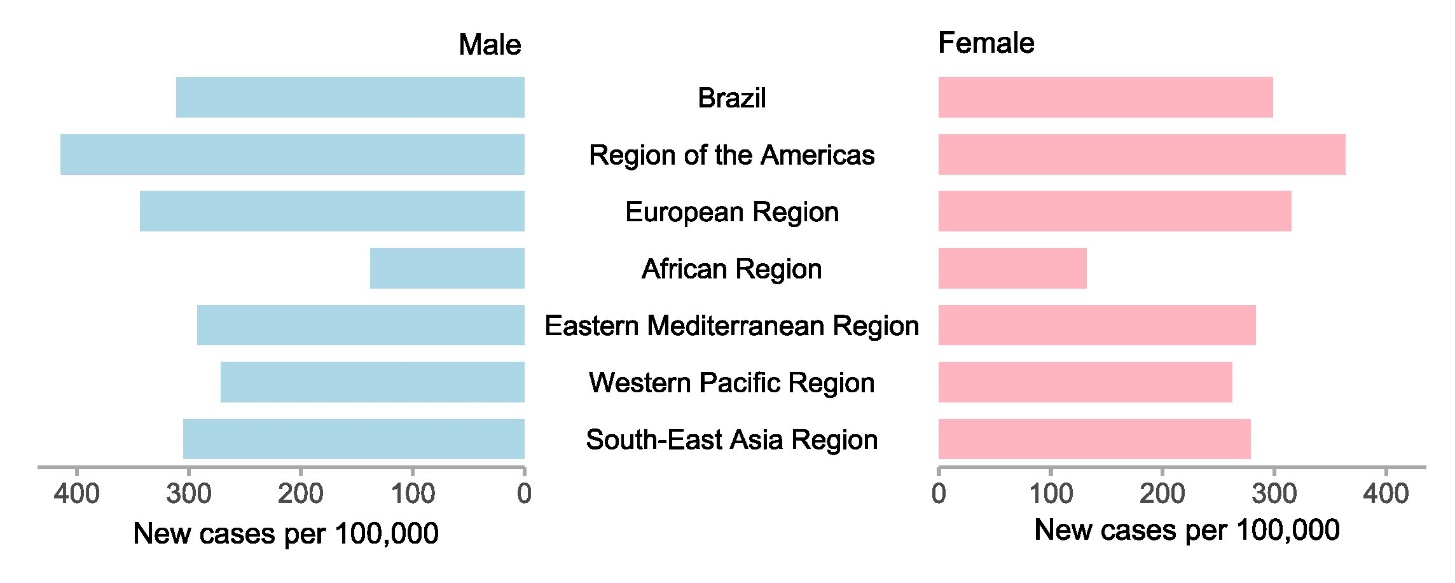

Supplement: Supplementary file 1 [file DataSheet_1.docx]
